# Metastatic Tumor of the Spermatic Cord in Adults: A Case Report and Review

**DOI:** 10.1155/2015/747261

**Published:** 2015-12-03

**Authors:** Daisaku Hirano, Mizuho Ohkawa, Ryo Hasegawa, Norimichi Okada, Naoki Ishizuka, Yoshiaki Kusumi

**Affiliations:** ^1^Department of Urology, Higashimatsuyama Municipal Hospital, Higashimatsuyama 355-0005, Japan; ^2^Department of Surgery, Higashimatsuyama Municipal Hospital, Higashimatsuyama 355-0005, Japan; ^3^Department of Pathology, Nihon University School of Medicine, Tokyo, Japan

## Abstract

Metastatic spermatic cord (SC) tumor is extremely rare. Recently, we experienced a case of late-onset metastatic SC tumor from cecal cancer. This case is a 68-year-old man presenting with a painless right SC mass. He had undergone a right hemicolectomy for cecal cancer 6 years ago. Radical orchiectomy and adjuvant chemotherapy with S-1 were performed. No recurrence was found after one year of follow-up. We identified a total of 25 cases, including our case, on a literature search via PubMed from January 2000 to April 2015. The most frequent primary sites of the tumors metastasizing to the SC were the stomach (8 cases, 32%) and the colon (8 cases, 32%), next the liver (2 cases, 8%), and kidney (2 cases, 8%). The majority of the cases underwent radical orchiectomy for the metastatic tumors of the SC. Over half of the cases received adjuvant interventions based on the regimens for the primary tumors. Prognosis in the patients with metastatic tumor of the SC was unfavorable except for late-onset metastasis. In patients with a mass in the SC and a history of neoplasm, especially in gastrointestinal tract, the possibility of metastasis from the primary cancer should be considered.

## 1. Introduction

Tumors arising from the spermatic cord (SC) are rare and most of these tumors are benign such as lipoma. However, approximately 25% are potentially life-threating malignant neoplasms [1]. The most common malignant tumors comprise sarcomas such as liposarcoma, leiomyosarcoma, rhabdomyosarcoma, and malignant fibrous histiocytoma and occur as a result of a mutation of a pluripotent mesenchymal cell that transforms into a malignant population clones [2].

On the other hand, metastatic SC tumor is even more unusual [3]. Several investigators have indicated that the most frequent primary tumors metastasizing to the SC and peritesticular tissues have been neoplasms of the stomach and prostate [4]. The timings of the detection of SC metastasis in most previous reports have been synchronous or metachronous, and the majority of cases in the metachronous were found in less than several years after the treatment for primary tumors [4]. Recently, we experienced a patient with cecal cancer recurrence in the SC that occurred on late phase after radical hemicolectomy. Herein, we report this case and a review of the recent literatures.

## 2. Case Report

**Figure 1:**
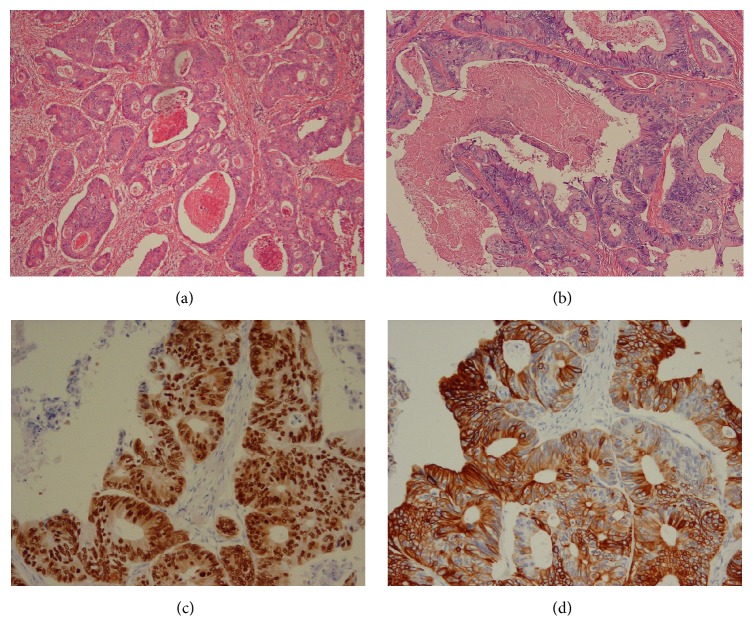
Histopathology. (a) Primary cecal cancer reveals moderately differentiated adenocarcinoma. (b) Spermatic cord tumor shows moderately differentiated adenocarcinoma, which is compatible with a metastasis from the cecal cancer. (c) Immunohistochemical staining indicates caudal-type homeobox- (CDX-) 2 positive in the spermatic cord tumor. (d) Immunohistochemical staining shows cytokeratin- (CK-) 20 positive in the spermatic cord tumor.

A 68-year-old man was referred to the Department of Urology from the Surgery at Higashimatsuyama Municipal Hospital with a right painless inguinal mass in April 2014. He had noticed it 6 months previously and observed an increase of its size. He had undergone right radical hemicolectomy for cecal cancer 6 years ago. Histological examination of the extirpated colon specimen showed moderately differentiated adenocarcinoma (Figure 1(a)) with depth of invasion of subserosa, lymphovascular invasion, and metastasis of the paracolic lymph nodes.

**Figure 2:**
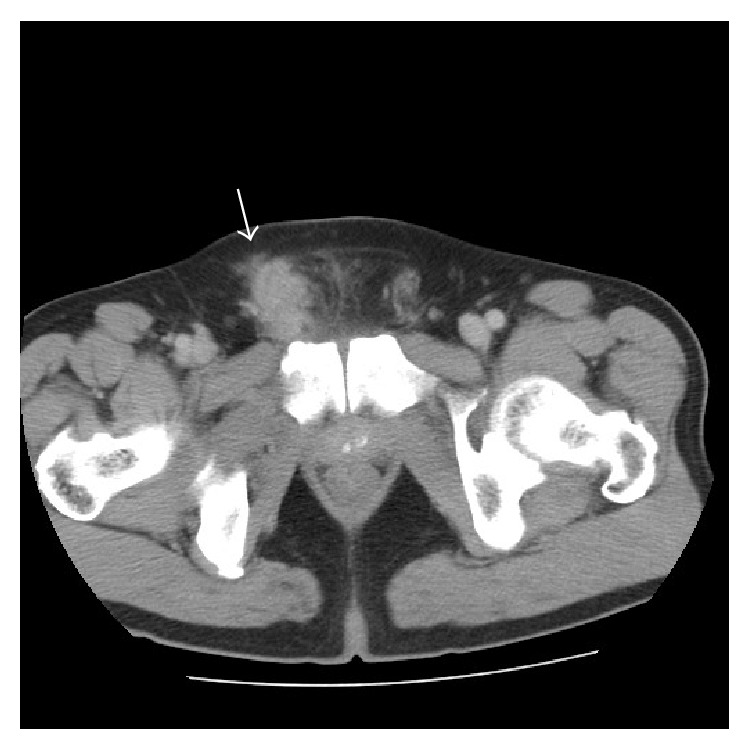
Abdominal CT. Abdominal CT reveals a 3.7 cm diameter slightly enhanced tumor (arrrow) in the right spermatic cord.

On physical examination, an approximately 4 × 3 cm palpable relatively fixed unpainful mass was observed in the right inguinal region. Abdominal computed tomography (CT) scan showed a heterogeneously slight-enhanced mass with noncapsulated irregular shape, 3.7 cm in diameter, suspicious of extending to the adjunct structures (Figure 2). Serum carcinoembryonic antigen (CEA) value was slightly elevated to 7.0 ng/mL (normal range < 5.0 ng/mL) but the levels of other tumor markers such as carbohydrate antigen 19-9 (CA19-9) and prostate-specific antigen (PSA) were normal. We did not deny that this tumor had been potentially malignant based on the clinical findings.

**Figure 3:**
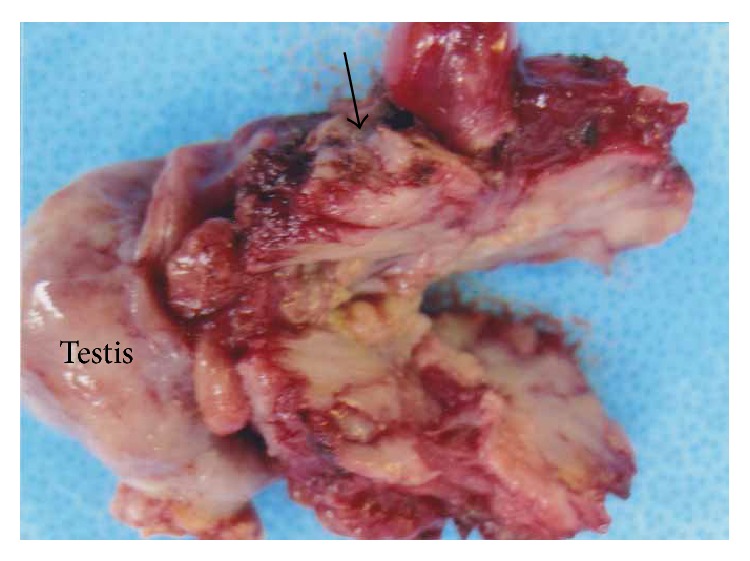
Gross appearance of the resected tumor. Gross examination shows grayish-white mass (arrrow) in the cut surface of the resected tumor.

The patient underwent right radical orchiectomy. During the operation, the tumor was located in the SC and showed relatively invasive growth to the adjunct structures but did not invade the epididymis and testis. The resected specimen involved a 4.5 × 3.5 × 3 cm solid mass, with grayish-white tumor in the cut surface, and was located in the lower part of the SC (Figure 3).

A histological examination of the SC tumor showed moderately differentiated adenocarcinoma (Figure 1(b)). In an immunohistochemical examination, the tumor cells in the SC were stained for caudal-type homeobox- (CDX-) 2 (Figure 1(c)) and cytokeratin- (CK-) 20 (Figure 1(d)). Based on the histopathological and immunohistochemical findings, the SC tumor was compatible with a metastasis from the cecal cancer.

The postoperative course was uneventful. The patient received adjuvant chemotherapy with tegafur/gimeracil/oteracil (S-1) which is a combined oral chemotherapeutic agent and has been doing well without evidence of recurrence for one year following the surgery.

## 3. Discussion

The SC is an extremely rare site for distant metastasis from a malignant neoplasm. In an autopsy study there were only two metastatic sites (0.01%) of the SC among 13,500 autopsy cases, both of which were from a primary gastric cancer [3]. The most common primary origin of a SC metastasis was the stomach, followed by the prostate, ileum, kidney, and colon as previously reviewed by Algaba et al. [4]. That review is over thirty years old and to our knowledge, there have not been any reviews involving recent cases. We performed a literature search of the case reports on adult metastatic SC tumor via PubMed from January 2000 to April 2015.  Table 1 shows identified cases [5–27] including our case since 2000. The mean age of incidence was 61 years (range: 36 to 85 years). The overall incidence was similar on both sites, but with respect to the colon cancers as the primary site the ascending colon and cecal cancers metastasized to the right SC while the descending colon and sigmoid cancers metastasized to the left SC. The most presenting symptoms were a mass in inguinal sites and scrotal swelling, both with and without pain except for one case that was incidentally found in an orchiectomy specimen due to the treatment of prostate cancer. The average metastatic tumor size of the SC in the identified 15 cases was 3.6 cm in a diameter (range: 1.6 to 6.5 cm). The most frequent primary origin of the tumor was the stomach (8 cases, 32%) and colon (8 cases, 32%), followed by liver (2 cases, 8%) and kidney (2 cases, 8%), and one case occurring in the small bowel, gastrointestinal tract, pancreas, lung, and prostate each. The average time between diagnosis of primary tumor and the presence of metastasis to the SC in the 15 metachronous cases (60%) was 42 months (range: 2 to 108 months), while seven cases (28%) were synchronously detected and three cases (12%) were found as an occult cancer. The metastatic SC tumors extending to the epididymitis were found in six (32%) of the identified 19 cases, of which two cases (11%) invaded the testis.

The vast majority of cases as well as our case underwent radical orchiectomy for the metastatic tumors in the SC, while tumor resection alone with preservation of the testis was found in two cases (8%). Over half of the cases received adjuvant interventions such as chemotherapy, molecular therapy, or hormone therapy based on the regimens for the primary tumors.

**Figure 4:**
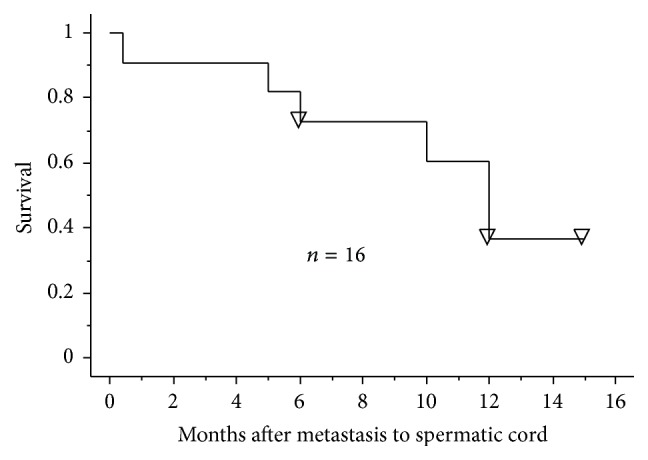
Survival after metastasis to the spermatic cord in the identified cases since 2000.

The prognosis of a metastatic tumor in the SC has been typically unfavorable as previously reported [4]. In this review, the 2-year survival rate in the postmetastasis to the SC was 36% in a total of the 16 patients identified since 2000, including our case, using the Kaplan-Meier method with a median follow-up duration of 12 months (range: 0.47 to 26 months) (Figure 4). Although there have been a small number of cases and short follow-up duration, the prognosis in patients with a metastatic SC tumor seems to be unfavorable even in the recent cases. However, the patients with late-onset (6 years or more) metastasis to the SC are likely to be a favorable prognosis because four (80%) of the five patients had been alive without recurrence after radical orchiectomy with a mean follow-up duration of 16 months.

The mechanisms of metastasis to the SC and paratesticular tissues from primary malignant neoplasms have not been precisely elucidated. However, several possibilities have been proposed. The main routes have been postulated to be vascular and lymphatic routes. Other routes involving retrograde extension through the vessel, either along its lumen or by direct extension via the wall of the vessel, and transperitoneal seeding through the patent tunica vaginalis have been proposed [3, 4]. In our case hematogenous or lymphatic spread may be possible due to the positive lymphovascular invasion as well as the evidence of the paracolic lymph nodes metastasis in the primary neoplasm, and the late recurrence after the treatment of the primary site may be related to the activation of long-lasting tumor dormancy in distant organs including the SC.

In conclusion SC solid masses are usually not considered as SC metastasis from primary neoplasms such as gastrointestinal tract cancers previously treated with a curative intention. We did not initially consider this case to be a metastasis from cecal cancer. However, in patients with solid mass of the SC and a history of neoplasm, especially in the gastrointestinal tract, and even though the primary neoplasm has been treated with a curative intent long time earlier, the solid mass of the SC should be kept in mind in a possibility of metastasis from the primary tumor.

## Figures and Tables

**Table 1:** Reports of metastatic tumors of the spermatic cord in adults since 2000.

Case number	Author (year)	Age	Site	Symptoms	Tumor size (SC) in diameter, cm	Primary site	Duration of detection of SC metastasis from primary site diagnosis	Histopathology (SC tumor)	Involved structure	Treatment (SC tumor)	Prognosis after treatment
1	Ota et al. [5] (2000)	51	Left	Painless scrotal swelling	2.5	Stomach	9 years	Poorly differentiated adenocarcinoma	Epididymis and parietal tunica vaginalis	Radical orchiectomy + chemo (MTX, 5-FU)	Died, 1 year

2	Polychronidis et al. [6] (2002)	63	Left	Painless scrotal swelling	2	Colon (sigmoid)	Occult	Mucus-secreting adenocarcinoma	Intact	Radical orchiectomy	ND

3	Bawa et al. [7] (2003)	85	Left	Incidentally found by castration	ND	Prostate	Synchronous	Adenocarcinoma	Vas deferens	Radical orchiectomy + hormone therapy	ND

4	Salesi et al. [8] (2004)	62	Left	Mass in scrotum	ND	Gastrointestinal tract	Occult	Adenocarcinoma	Epididymitis	Orchifunicolectomy + chemo (CDDP, Epirubicin, 5-FU)	Died, 5 months

5	Bandyopadhyay et al. [9] (2005)	67	Right	Mass in groin	ND	Pancreas	Synchronous	Moderately differentiated adenocarcinoma	ND	Radical orchiectomy + distal pancreatectomy	ND

6	Kaya et al. [10] (2006)	62	Left	Painful mass in inguinal site	4.5	Lung	Synchronous	Non-small cell adenocarcinoma	Intact	Radical orchiectomy	Died, 2 weeks

7	Shida et al. [11] (2006)	75	Left	Mass in inguinal site	5	Colon (ascending)	2 months	Poorly differentiated adenocarcinoma	Intact	Radical orchiectomy	Died, 6 months

8	Miyake et al. [12] (2007)	60	Right	Mass in inguinal site	3	Colon (ascending)	1 year and 8 months	Moderately differentiated adenocarcinoma	Intact	Radical orchiectomy	ND

9	Paravastu et al. [13] (2007)	62	Left	Painless scrotal swelling	ND	Colon (descending)	Synchronous	Poorly differentiated adenocarcinoma	Intact	Radical orchiectomy + chemo (irinotecan, fluorouracil, cetuximab)	Alive, 18 months

10	Galanis et al. [14] (2009)	80	Right	Painful mass in inguinal site	ND	Colon (cecum, ascending, sigmoid)	Synchronous	Adenocarcinoma	Intact	Radical orchiectomy	Died, early postoperative period

11	Chang et al. [15] (2009)	38	Right	Scrotal enlargement and chronic testicular pain	ND	Liver	7 months	Cholangiocarcinoma (Klatskin tumor)	ND	Biopsy of the spermatic cord tumor careful surveillance	Alive, 5 months

12	Correa et al. [16] (2009)	57	Left	Mass in inguinal site	5	Left kidney	Synchronous	Renal cell carcinoma clear cell type	ND	Radical orchiectomy + radical nephrectomy + Sunitinib	Alive, 1 year

13	Schaefer et al. [17] (2010)	64	Right	Painful mass in groin and scrotum	2	Stomach	Synchronous	Signet ring cell carcinoma	Epididymitis and testis	Radical orchiectomy + chemo (paclitaxel, leucovorin, 5-FU: FLF regimen)	Died, 1 year

14	Ishibashi et al. [18] (2011)	71	Right	Mass in groin	3.8	Colon (cecum)	1 year	Well-differentiated adenocarcinoma	Intact	Radical orchiectomy + chemo (S-1)	Alive, 15 months without recurrence

15	Chiang et al. [19] (2011)	57	Right	Painful hard mass in inguinal site	2	Liver	6 years	Hepatocellular carcinoma	Intact	Radical orchiectomy + adjuvant radiotherapy	Alive, 6 months without recurrence

16	Mohammadi et al. [20] (2011)	57	Left	Painless mass in high scrotal site	6.5	Left kidney	3 years	Renal cell carcinoma (clear cell type)	ND	Tumor resection with preserved testis	Alive, 3 months without recurrence

17	Al-Ali et al. [21] (2012)	77	Left	Inguinal and testicular pain	ND	Colon (descending)	2.5 years	Adenocarcinoma	Epididymitis and capsule of the testis	Radical orchiectomy	ND

18	Lee et al. [22] (2012)	57	Left	Mass in inguinal site	4	Stomach	3 years	Poorly differentiated adenocarcinoma	Intact	Radical orchiectomy	ND

19	Watanabe et al. [23] (2013)	52	Right	Mass in inguinal and scrotal site	2	Stomach	2 years	Poorly differentiated adenocarcinoma	ND	Radical orchiectomy + chemo (CDDP)	Died, 10 months

20	Valizadeh et al. [24] (2013)	36	Right	Painful mass in inguinal site	1.6	Small bowel	Occult	Adenocarcinoma	ND	Radical orchiectomy + small bowel tumor resection + chemo (capecitabine plus oxaliplatin regimen)	Alive, 6 months without recurrence

21	Kanazawa et al. [25] (2013)	66	Right	Groin pain	4.2	Stomach	1 year	Moderately differentiated tubular adenocarcinoma	ND	Tumor resection with preserved testis + chemo	ND

22	Xu and Wang [26] (2013)	50	Bil	Mass in spermatic cords	ND	Stomach	4 years	Signet ring cell carcinoma	Epididymitis and seminiferous duct	Radical orchiectomy	ND

23	Kim et al. [27] (2014), Case 1	49	Right	Mass in scrotum with discomfort in spermatic cord	4	Stomach	7 years	Mucinous adenocarcinoma with signet ring cell carcinoma	Epididymitis	Radical orchiectomy + chemo (folinic acid, fluorouracil, oxaliplatin; FOLFOX regimen)	Alive, 26 months without recurrence

24	Case 2	60	Left	Inguinal pain	3.5	Stomach	6 years	Mucinous moderately differentiated adenocarcinoma	Intact	Radical orchiectomy + adjuvant radiation	Alive, 20 months without recurrence

25	Present study	68	Right	Painless mass in inguinal site	4.5	Colon (cecum)	6 years	Moderately differentiated adenocarcinoma	Intact	Radical orchiectomy + chemo (S-1)	Alive, 12 months without recurrence

S-1: tegafur/gimeracil/oteracil.

ND: Not document, SC: Spermatic cord.
